# Supplementary material for: Implication of TIGIT+ human memory B cells in immune regulation
Source: Nat Commun. 2021 Mar 9;12:1534. doi: 10.1038/s41467-021-21413-y (PMC7943800; doi:10.1038/s41467-021-21413-y)
Supplement: Supplementary file 1 — Supplementary information [file 41467_2021_21413_MOESM1_ESM.pdf]

SUPPLEMENTARY INFORMATION

Implication of TIGIT+ human memory B cells in immune regulation

Md Mahmudul Hasan[1,] Sumi Sukumaran Nair[2], Jacqueline G O'Leary[3], Luan Snipes-Thompson[3], Verah Nyarige[4], Junwen Wang[4], Walter Park[5], Mark Stegall[5], Raymond Heilman[2], Goran B. Klintmalm[3], HyeMee Joo[1*] and SangKon Oh[1*]

[1]Department of Immunology, Mayo Clinic, Scottsdale, AZ 85259, USA.
[2]Department of Medicine, Mayo Clinic, Phoenix, AZ 85054, USA
[3]Baylor University Medical Center, Dallas, TX 75246, USA.
[4]Department of Health Sciences Research, Mayo Clinic, Scottsdale, AZ 85259, USA.
[5]William J. von Liebig Transplant Center, Mayo Clinic, Rochester, MN 55905, USA

This file contains:
Supplementary Figs. 1-16
Supplementary Tables 1-3

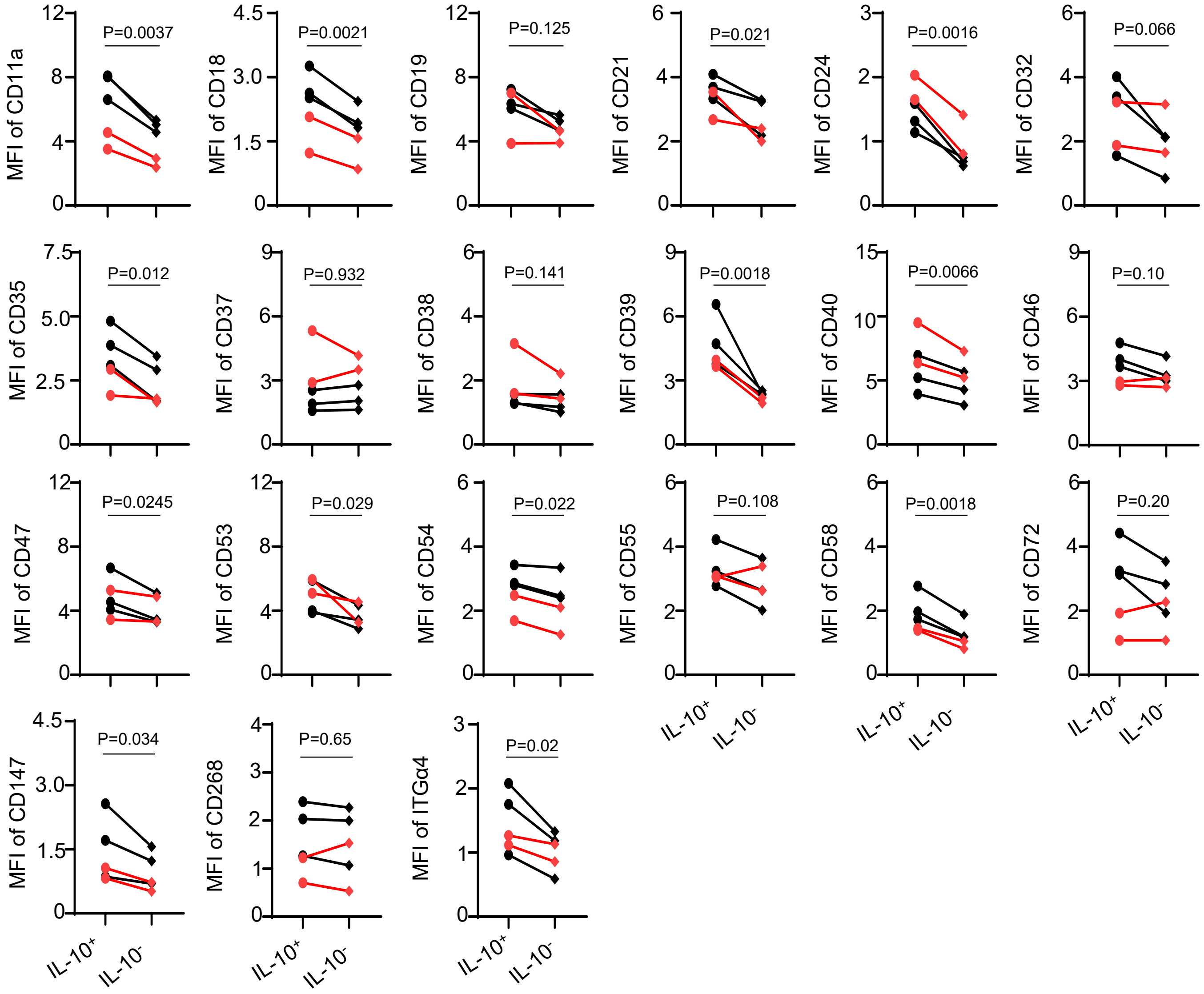

Supplementary Fig. 1 Surface phenotypes of IL-10^{+} and IL-10^{-} B cells.
B cells from the blood of healthy subjects (red) and tonsillar B cells (black) were stimulated as in Fig. 1. Delta MFI values (1x10^{3}) of CD11a, CD18, CD19, CD21, CD24, CD32, CD35, CD37, CD38, CD39, CD40, CD46, CD47, CD53, CD54, CD55, CD58, CD72, CD147, CD268, and integrin α4 on both IL-10^{+} and IL-10^{-} B cells are presented. Each dot represents data generated with cells from different donors (n=5). Statistical significance was tested by a two-tailed paired *t*-test.

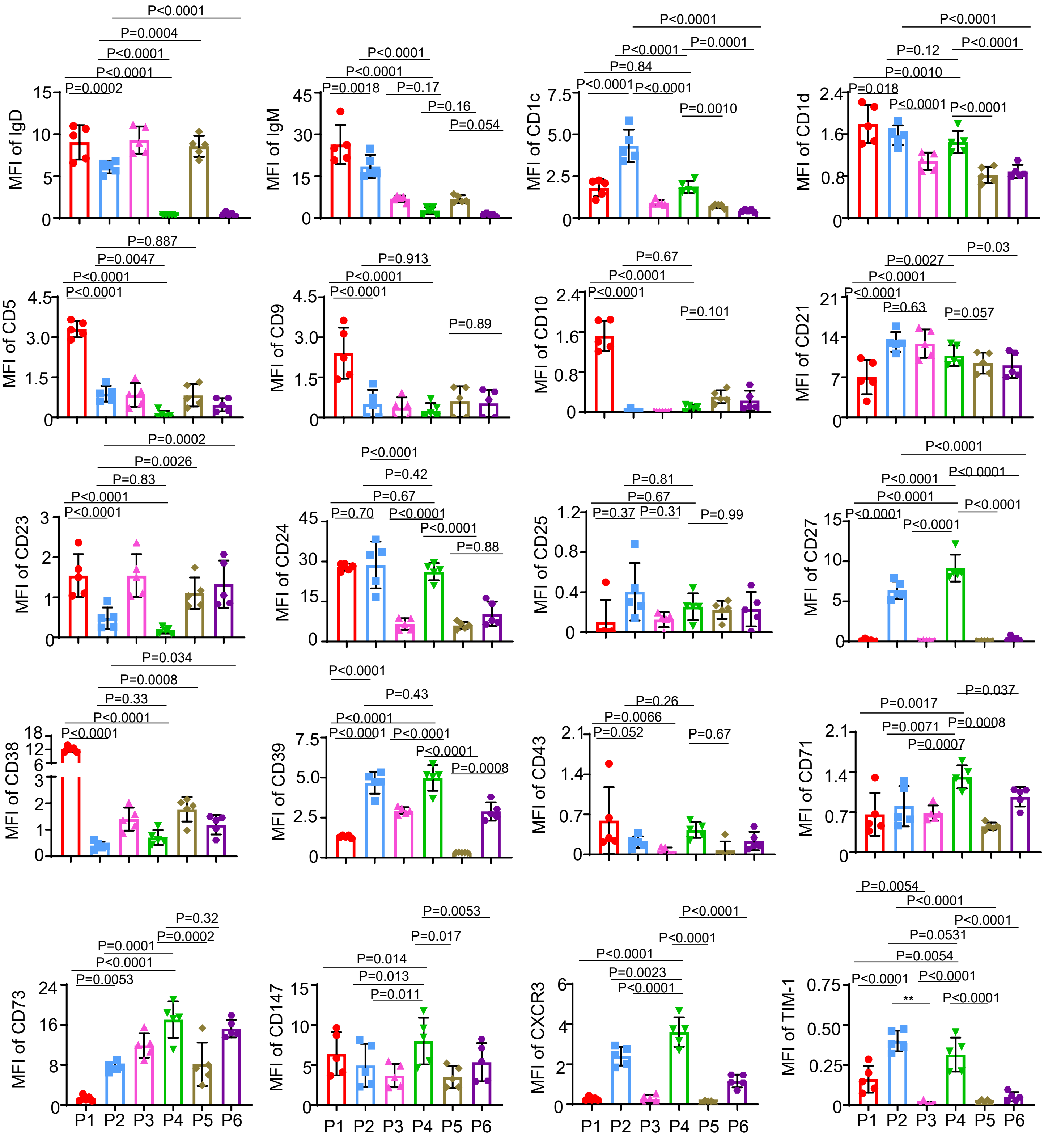

Supplementary Fig. 2 Summary of MFI (1x10³) values of cell surface molecules tested in Fig. 2c. Individual dots represent data generated with cells from different donors (n=5). Error bars are mean ± SD. One-way ANOVA with Holm-Sidak's multiple comparisons test was used.

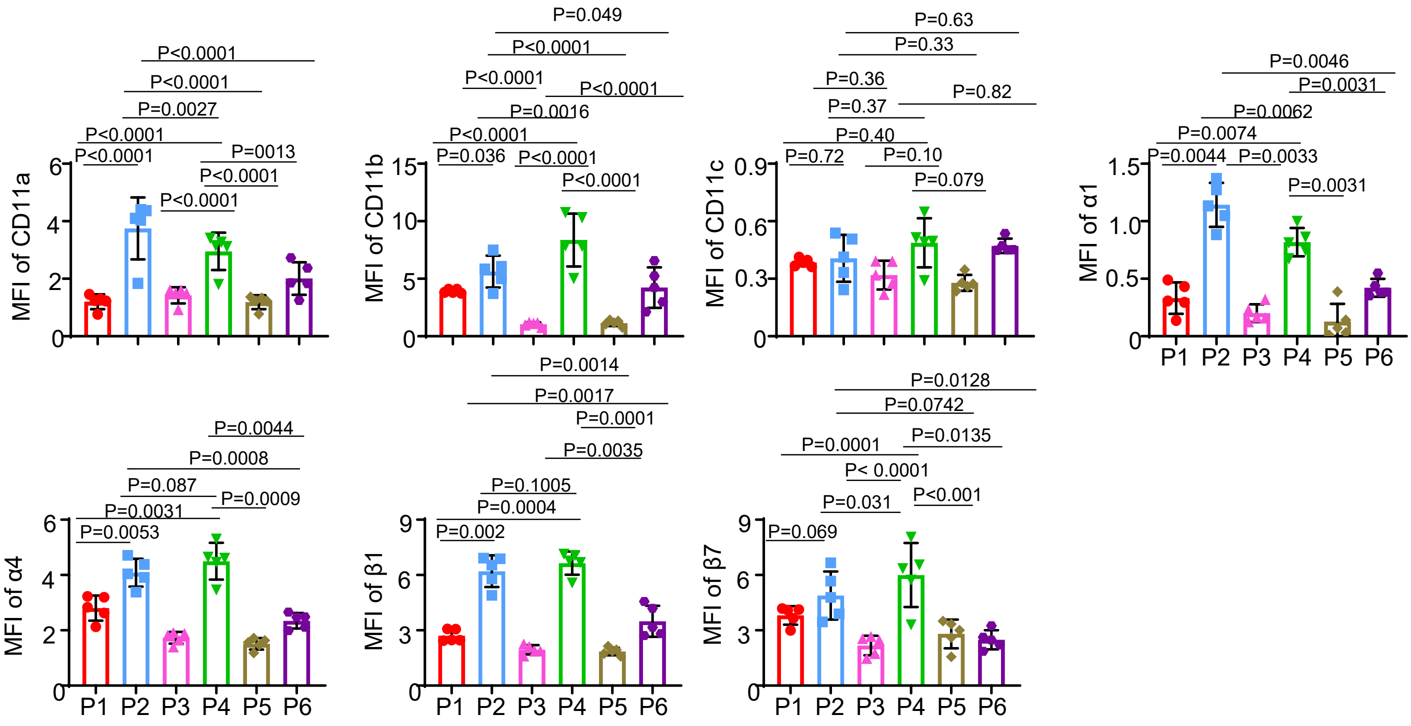

Supplementary Fig. 3 Summary of MFI (1x10^3) values of cell integrins tested in Fig. 2c. Individual dots represent data generated with cells from different donors (n=5). Error bars are mean ± SD. One-way ANOVA with Holm-Sidak's multiple comparisons test was used.

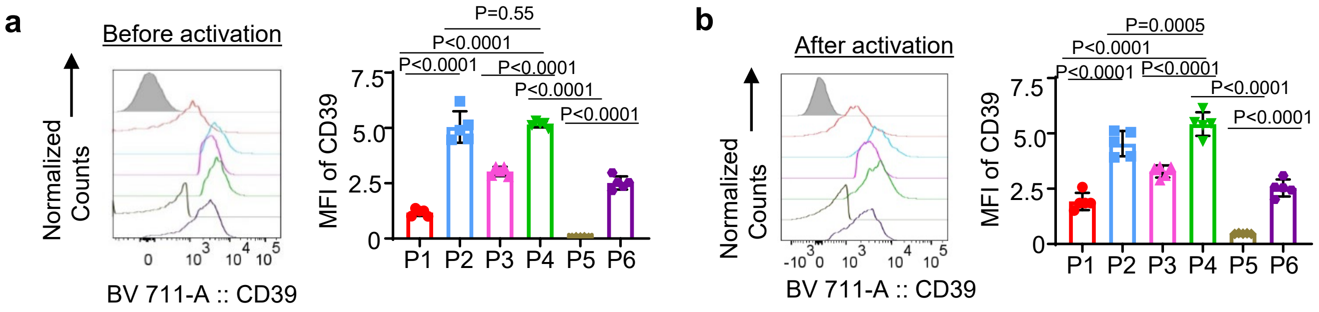

Supplementary Fig. 4 CD39 expression on P1-P6 B cells before and after activation with CpG-B. (a-b) FACS plot and bar graphs showing MFI values ($1x10^3$) of CD39 on P1-P6 B cell subsets before (a) and after 16 hrs CpG-B activation (b). Individual dots in **a** and **b** represent data generated with cells from different donors (n-=5). Error bars are mean ± SD. P values were acquired with one-way ANOVA with Holm-Sidak's multiple comparisons test.

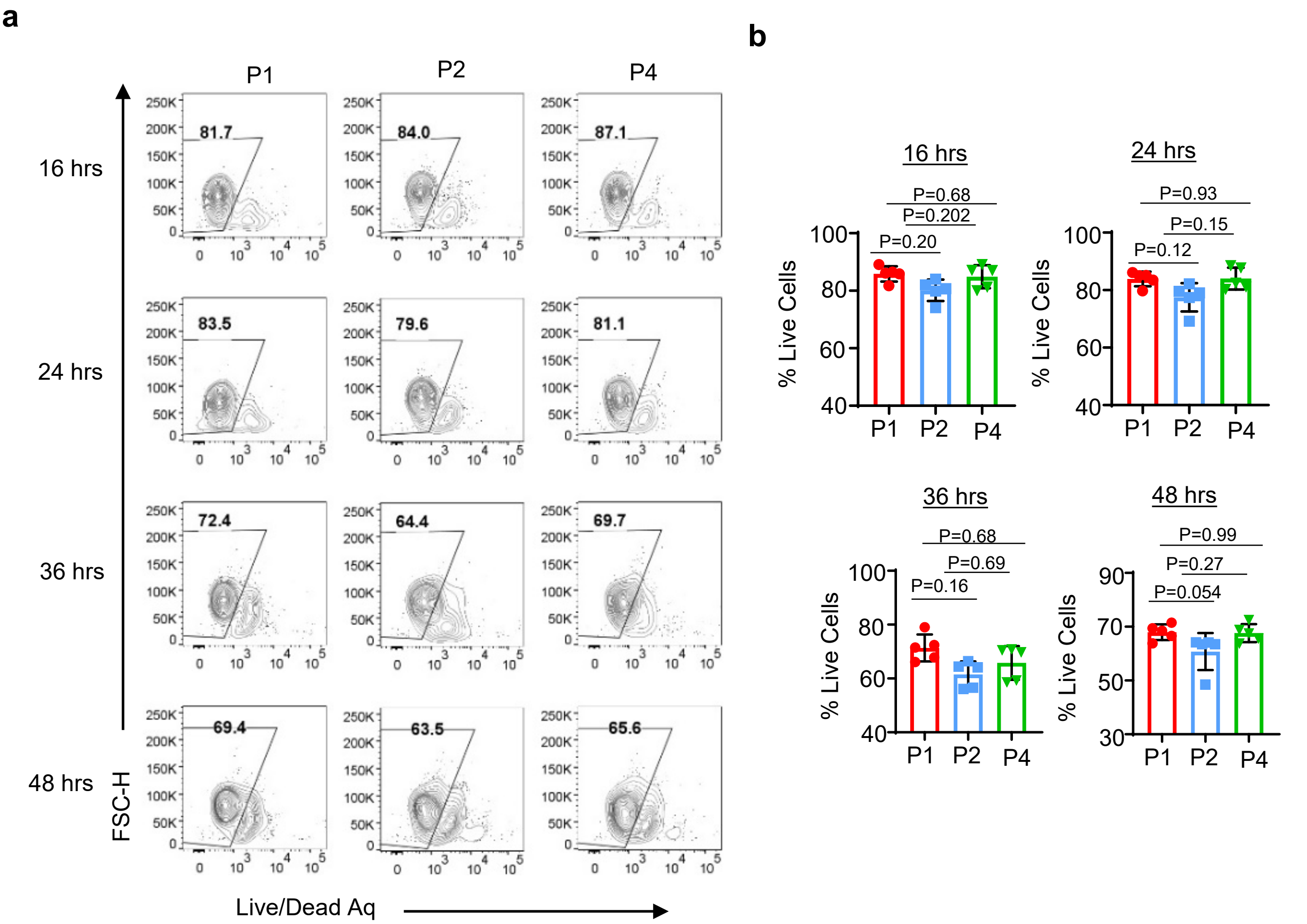

Supplementary Fig. 5 P1, P2, and P4 B cells maintain similar cell viability during *in vitro* culture. FACS-sorted P1, P2, and P4 B cells were cultured in the presence of CpG-B for 48 hrs. The frequency of live and dead cells were assessed using Live/Dead Fixable Aqua Dead Cell Stain Kit. (a) Representative FACS data are presented. (b) Summarized data generated with cells from the blood of different healthy subjects (n=5) in three independent experiments. Error bars are mean ± SD. P values were acquired with one-way ANOVA with Holm-Sidak's multiple comparisons test.

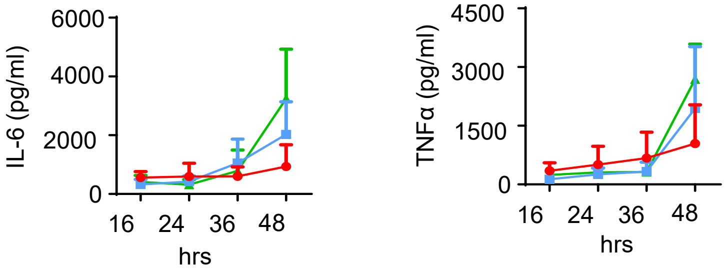

Supplementary Fig. 6 P1, P2, and P4 B cells can express both IL-6 and TNFα in response to CpG-B stimulation. FACS-sorted P1, P2, and P4 B cells were cultured in the presence of CpG-B for 48 hrs as in Fig.3d. Culture supernatants were collected at indicated time points. The amount of IL-6 and TNFα in the supernatants were assessed with bead-based multiplex assays. Summarized data of from three independent experiments using cells from healthy subjects (n=4). Error bars are mean ± SD.

a

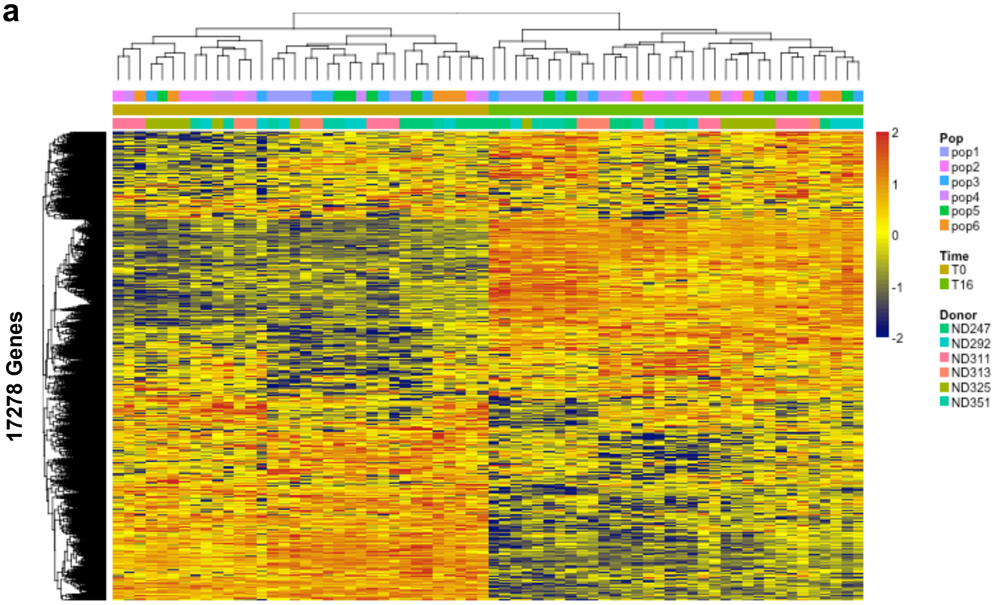

b

Comparison	FDR p < 0.05	FDR p < 0.01	FDR p < 0.05	FDR p < 0.01
	T0		T16	
Pop2 vs. Pop1	659	635	573	535
Pop2 vs. Pop4	116	91	80	57
Pop4 vs. Pop1	720	695	818	789
Pop2 vs. Pop356	297	274	241	213
Pop4 vs. Pop356	235	176	473	456
Pop1 vs. Pop356	413	386	220	191

Supplementary Fig. 7 Transcriptome analysis of FACS-sorted blood circulating B cell subsets (P1-P6). (a) Heatmap showing expression of all genes (17278) for individual donors before and after activation with CpG B. (b) Numbers of significantly expressed genes with FDR value P<0.05 among different B cell subsets. . One-way Welch analysis of variance (ANOVA) was conducted using a P-value cutoff of 0.05 and Benjamini-Hochberg multiple testing correction.

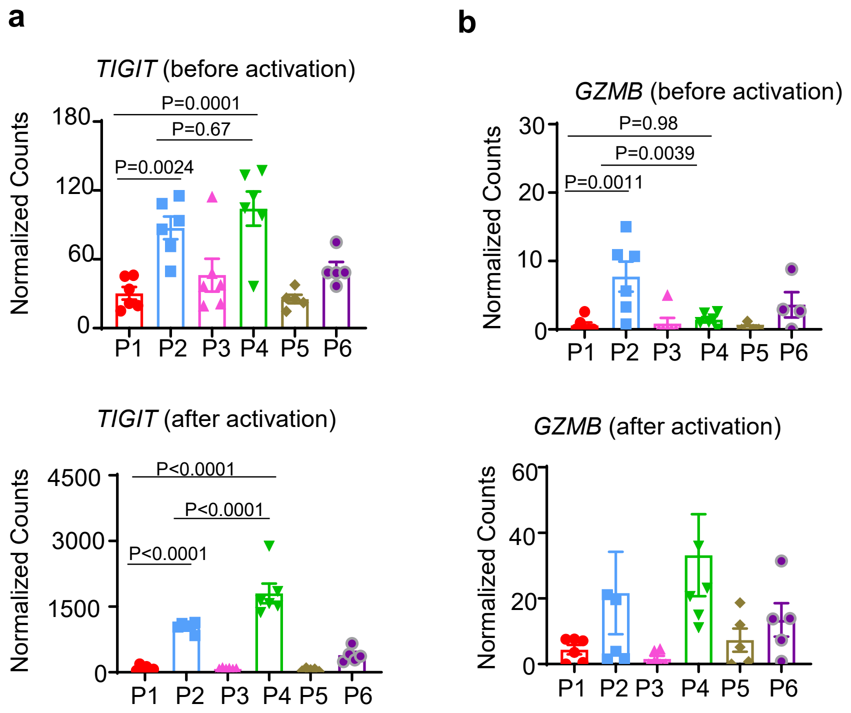

Supplementary Fig. 8 Normalized counts of *TIGIT* (a) and *GZMB* (b) expression before and after activation with CpG-B for 16 hrs. Error bars are mean ± SD. Data from six independent experiments performed with cells from healthy subjects (n=6). Statistical significance was tested with one way ANOVA with Holm-Sidak's multiple comparisons test.

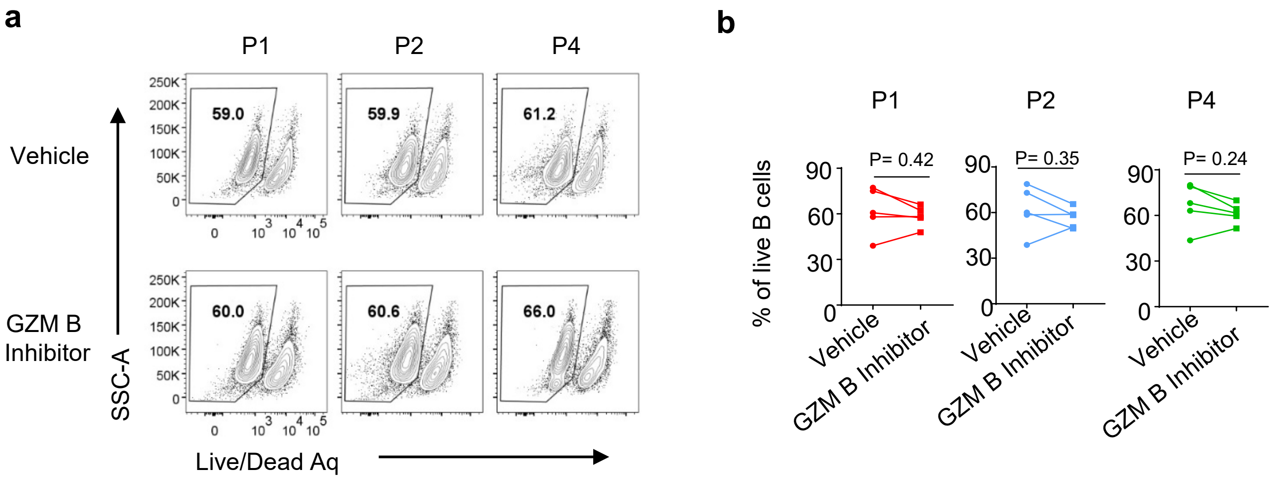

Supplementary Fig. 9 Granzyme B inhibitor does not alter B cell viability. B cells in Fig. 4f were stained with Live/Dead Fixable Aqua Dead Cell Stain Kit (a).Summarized data generated with cells from healthy subjects (n=5) in three independent experiments (b). Statistical significance was tested with a two-tailed paired *t*-test.

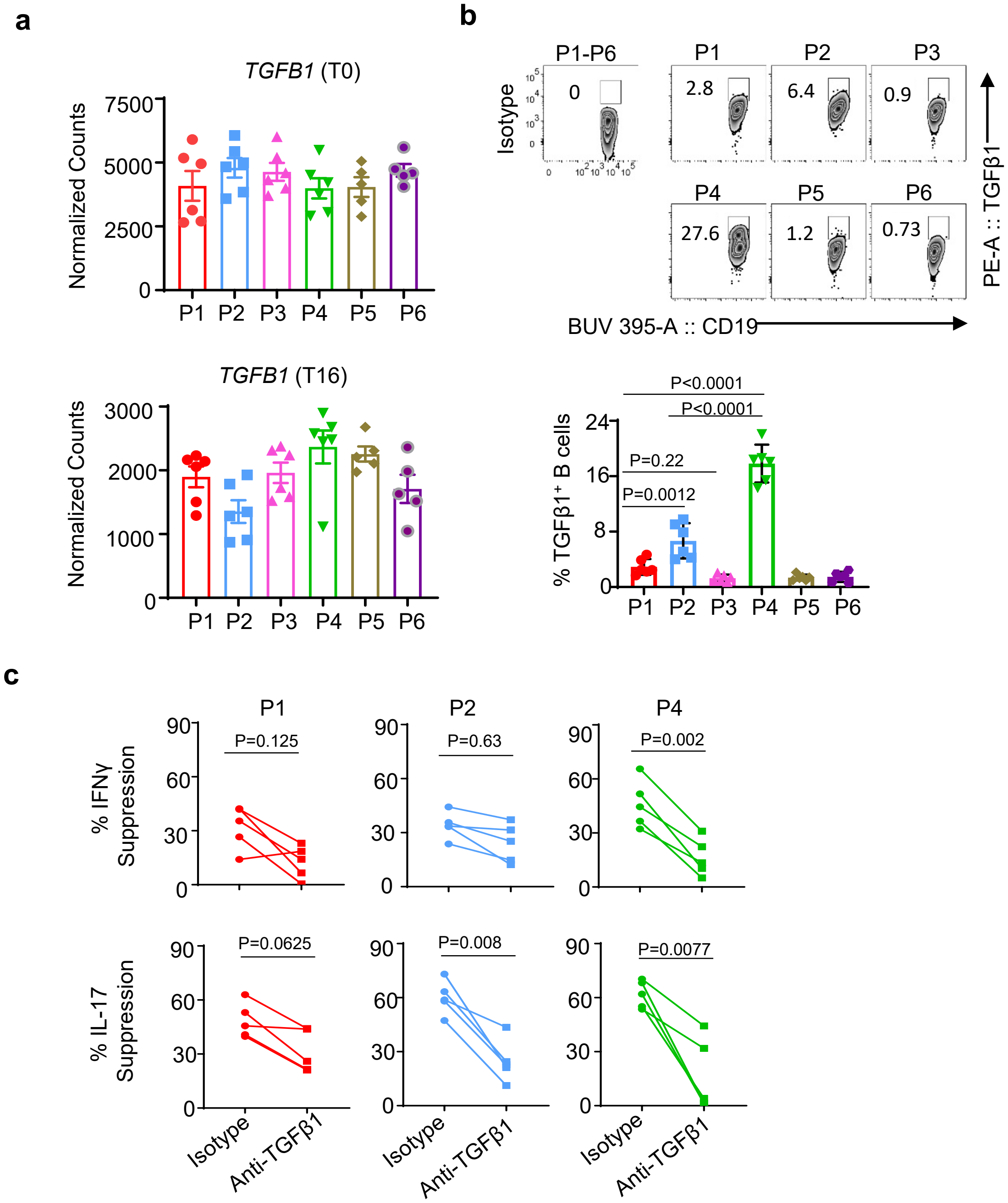

Supplementary Fig. 10 TGFβ1 expressed by P4 memory B cell can suppress IFNγ and IL-17 expression by CD4⁺ T cells . (a) Normalized counts of *TGFB1* before activation (upper panel) and after activation for 16 hrs with CpG-B (lower panel). RNA-Seq analysis for all six populations (P1-P6) purified from the blood of healthy individuals (n=6) was performed at once. (b) Representative FACS plots and summarized data showing TGFβ1 expression on P1-P6 B cell subsets upon 48 hrs CpG-B stimulation. Cells were further stimulated for 5 hrs with PMA/ionomycin in the presence of monensin and brefeldin A before staining them with anti-CD19 followed by intracellular TGFβ1 staining. Data were obtained from three independent experiments using cells from healthy donors (n=6). Error bars represent means ± SD. Statistical significance was tested with One-Way ANOVA with Holm-Sidak's multiple comparisons test . (c) Summarized data showing that blocking TGFβ1 with anti-TGF β1 antibody results in the recovery of both IFNγ and IL-17 expression by CD4⁺ T cells. FACS-sorted P1, P2, and P4 B cells were stimulated for 48 hrs with CpG-B, pre-incubated with anti-TGFβ1 or isotypes and then co-cultured for 4 days with autologous CD4⁺ T cells stimulated with anti-CD3/anti-CD28 beads. PMA, ionomycin, brefeldin A, and monensin cocktails were added 5 hrs before intracellular cytokine staining. Data from three independent experiments performed with cells from healthy individuals (n=5). Error bars are mean ± SD. Statistical significance was tested with a two-tailed paired *t*-test.

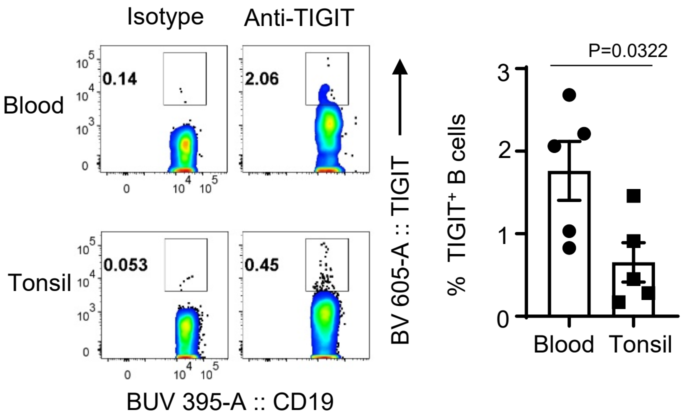

Supplementary Fig. 11 TIGIT expression on total CD19⁺ B cells from the blood and tonsils. Frequency of TIGIT⁺ B cells in the blood and tonsil was assessed. Individual dots represent data generated with cells from different donors (n=5) in three independent experiments. Error bars indicate mean ± SD. Statistical significance was tested with a two-tailed *t*-test.

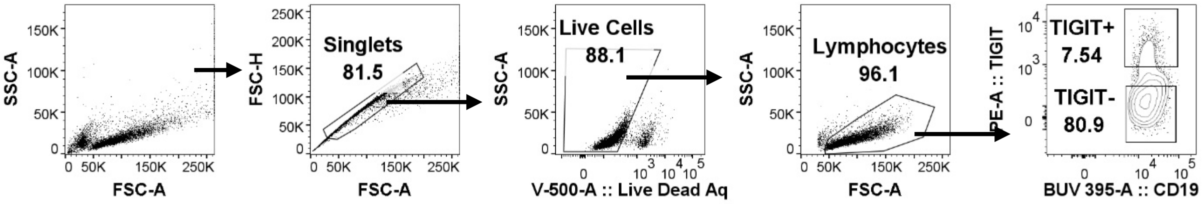

Supplementary Fig. 12. Gating strategies for TIGIT^{+} memory and TIGIT^{-} B cell sorting. Purified CD19^{+} memory B cells were stimulated with CpG-B for 48 hours. Cells were labeled with Live/Dead Aqua, anti-CD19, and anti-TIGIT prior sorting.

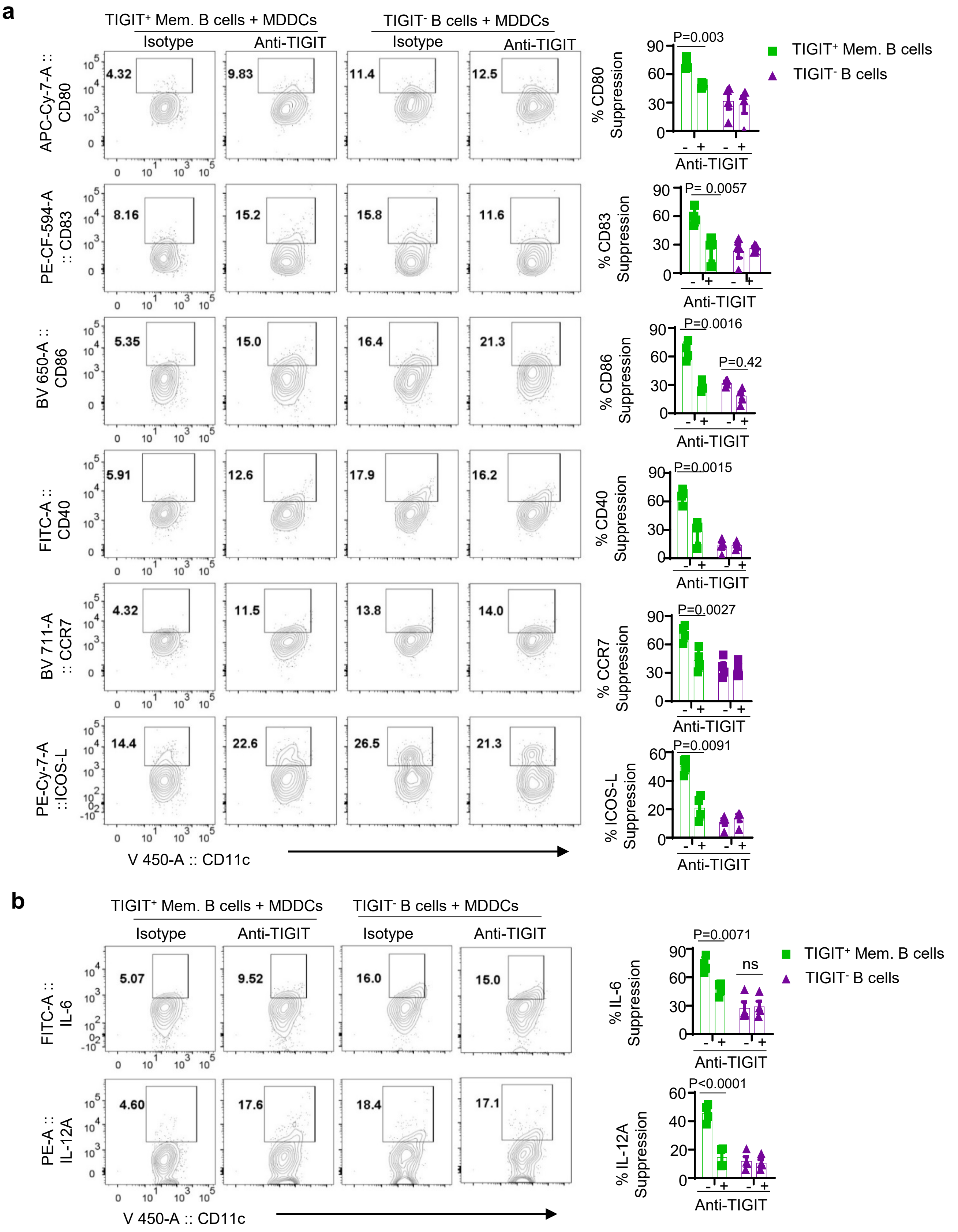

Supplementary Fig. 13 TIGIT expressed by memory B cells suppress DC maturation and proinflammatory cytokine expression . Representative FACS plots and summarized data showing the suppression of maturation markers (a) and proinflammatory cytokines (b) expression by DCs. LPS-stimulated MDDCs were cultured with or without TIGIT⁺ B cells or TIGIT⁻ B cells in the presence or absence of anti-TIGIT antibody. Fig. a and b, data were obtained from four independent experiments using cells from healthy donors (n=4). Error bars are mean ± SD. Statistical significance was tested by two-way ANOVA with Sidak's multiple comparisons test.

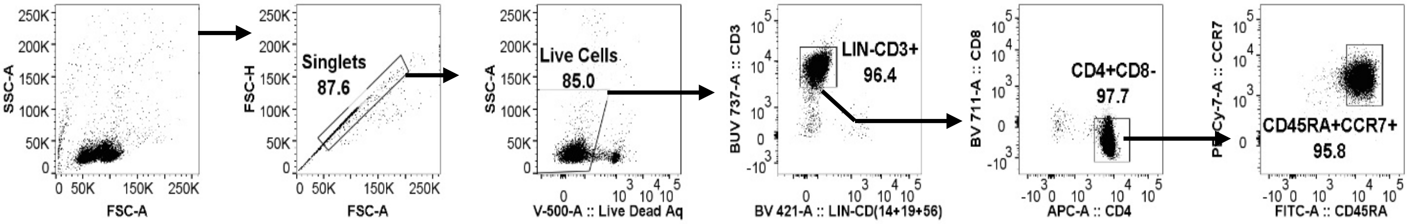

Supplementary Fig. 14. Gating strategies for naïve CD4⁺ T cell sorting. (a) Naïve CD4⁺ T cells were enriched with an enrichment kit. Cells were labeled with Live/Dead Aqua, anti-CD3, anti-CD8, anti-CD4, anti-CCR7, anti-CD45RA, and anti-(CD14, CD19, CD56) cocktails prior to cell sorting.

.

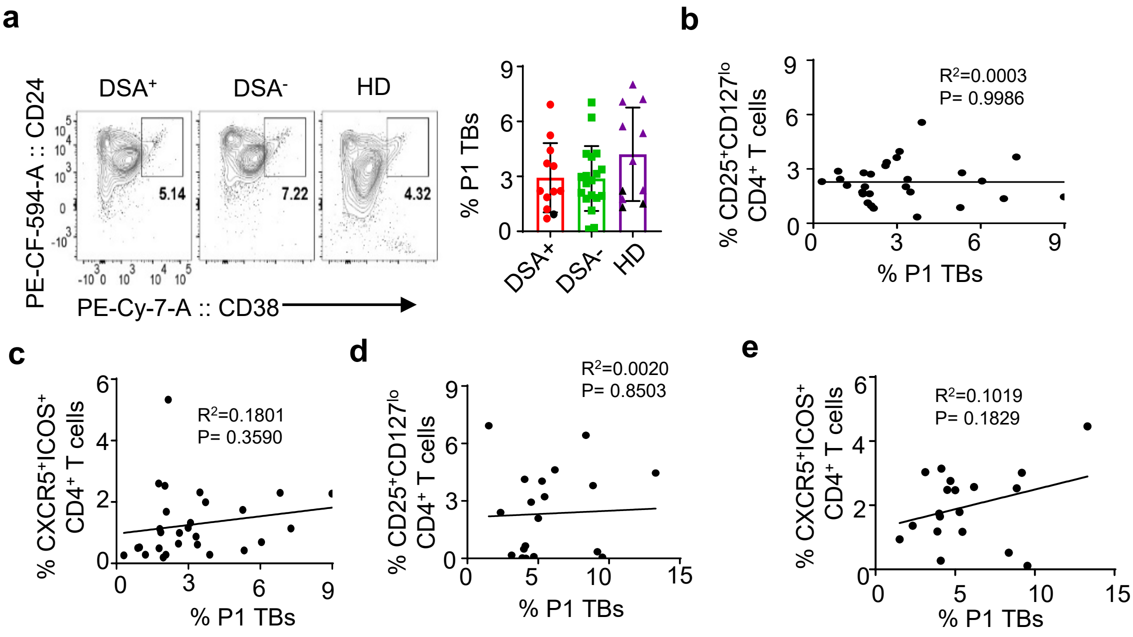

Supplementary Fig. 15. P1 TBs in liver (a-c) and kidney allograft recipients (d-e) in Fig. 7. (a) Representative FACS plots and summarized data for the frequency of $CD24^{hi}CD38^{hi}$ P1 TBs in the blood of DSA^{+} (n=12) and DSA^{-} (n=19) liver allograft recipients and healthy subjects (n=9). Dots represent individual patients. Bars indicate mean ± SD. (b) Correlation between the frequency of P1 TBs and that of Tregs in the blood of liver transplant patients. (c) Correlation between the frequency of P1 TBs and that of TFH cells in the blood of liver transplant patients. (d) Correlation between the frequency of P1 TBs and that of Tregs in the blood of renal allograft recipients. (e) Correlation between the frequency of P1 TBs and that of Tregs in the blood of renal allograft recipients. Individual dots represent data generated with cells from different donors. Pearson's correlation test was used.

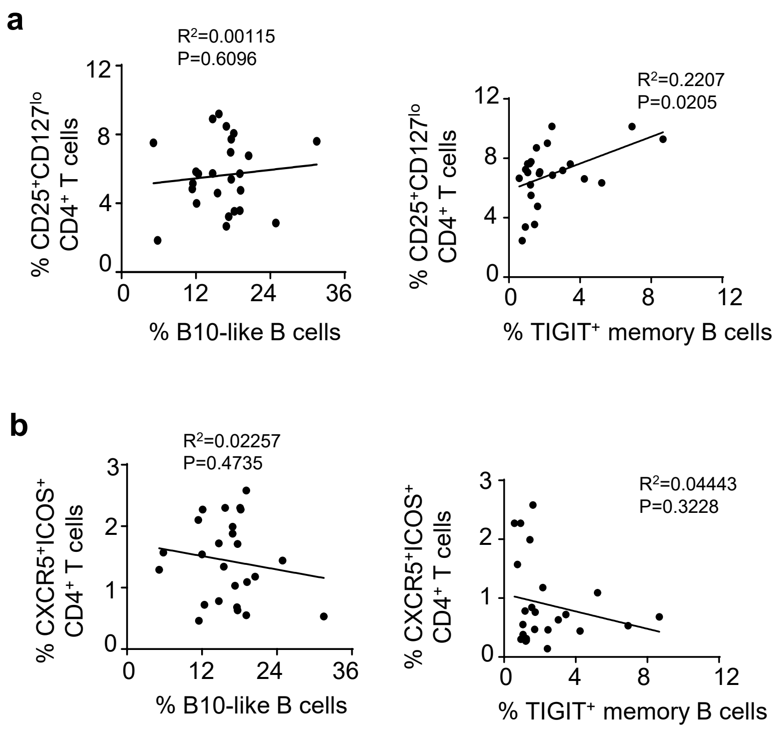

Supplementary Fig. 16. Analysis of the frequency of B cell subsets, CD25$^+$CD127^{lo} CD4$^+$ T cells , and CXCR5$^+$ICOS$^+$ CD4$^+$ T cells in the blood of healthy subjects. (a) Correlation between the frequency of CD25$^+$CD127^{lo} CD4$^+$ T cells and B cell subsets (n=17). (b) Correlation between the frequency of CXCR5$^+$ICOS$^+$ CD4$^+$ T cells and B cell subsets (n=17). Individual dots represent data generated with cells from different donors. Pearson's correlation test was used.

Supplementary Table 1: List of genes related to cell surface markers

PAM	P2RX4	EVA1C	CD19	PCDHGA11	CD300A
NTRK2	VSIG1	TRPV2	TTYH3	MFSD6	CD46
CD72	PCDHGA10	CD44	TNFRSF25	DEF6	GRIK4
LRP11	KISS1R	GPR25	ATRAID	FLT1	ROBO1
IL21R	PMEPA1	ATRNL1	CD53	SLC26A6	TPCN1
PCDH9	PCDHGB4	TMX4	CLDND1	GPER1	NTNG2
HFE	CCR10	DCBLD1	SLC16A1	F3	EMB
MUC16	PCDHGA8	MCAM	ITGAM	ITGAL	ERMP1
NCR3LG1	GPR18	IL12RB1	GPC2	SLC1A1	ZDHHC5
SLC37A4	TSPAN2	MFSD5	RIF1	ITGAV	CD59
MMP17	IL15RA	CD58	RRP1	CD1A	LPAR2
ADAM22	CR1	HCRTR1	EPHA4	VSIG10L	TMED7
PCDHGB2	SYPL1	ENG	LRP8	TNFSF13B	SLC6A3
PTAFR	LTB4R2	TMEM87B	MANSC1	HYAL2	ENSG00000116957
CHRNA6	PCDHGA6	TREML2	OPN3	TLR10	ENTPD1
GPRC5D	ZP3	PATJ	GLRA3	CD82	CXCR3
SDC1	PCDHGA4	GPR146	SLC40A1	SCN1B	QSOX2
SCN4A	SLC15A4	GPR34	PECAM1	EVI2A	SLC44A1
SLC19A1	BTNL9	ATP2B4	CD86	CHODL	EMP1
F2RL1	ABCB9	CCR1	NCR3	PCDHGA5	ASTN2
LAIR1	CSF2RB	SLC52A2	SGCE	FCRL5	NCAM2
KIAA1324	PCDHGB7	TMEM25	CD226	SLC1A2	CUZD1
CYSLTR1	CRB3	CLEC17A	SLC7A1	LIG3	FLVCR1
APLP2	EPHB1	ITGA3	ECE1	ABCG2	GUCY2C
ILDR1	GGT1	SLCO3A1	PLXNB1	CD80	BTN3A1
MBP	IL6R	TMEM67	MET	GPR55	TMEM150A
SIGIRR	F2RL3	RET	RNF149	LRIG1	PLD5
IL5RA	ABCB4	TMEM219	TBXA2R	GJA3	NTNG1
ESAM	FOLR2	SLC4A8	IL18R1	TMEM87A	ABCC4
ANTXR2	BTN1A1	BTN3A3	CD27	SLC17A9	PLXND1
CPD	SSPN	CD200	PTPRK	KLRG1	APP
KIAA0319	ENPP1	TLR6	SLC2A9	GINM1	WDR11
TNFRSF19	TNFRSF17	FAS	TAS1R3	EBP	SLC33A1
PSEN2	RHBDF2	IGHEP1	ABCA1	HLA.A	SLC2A1
TMEM154	ACVR1	TSHR	PTGIR	TNFRSF10A	SLC38A5
PCDHGB5	ENPP5	SLC23A2	C11orf24	SLC43A1	HTR1F
OR2C1	GDPD5	SLC10A3	TGOLN2	GPR68	ALCAM
NRN1	JAM2	PIGT	HM13	IL4R	SPRN
PCDHGB3	SHISA8	GPR108	MFSD2A	SLC7A6	CEACAM21
CLEC2D	EVI2B	TPRA1	SLC9A7	BMPR1A	NAGPA
ATP1B1	PTPRB	MPZL3	LILRA1	SGCB	SLC39A8
CNNM4	ITGB1	TSPAN5	PARM1	SLC4A7	DPEP3
CD5	BRAP	GPC4	TNFRSF13B	RPN1	SERINC3
TIGIT	CHRNA1	DCHS1	IL27RA	IFNLR1	SLC39A6
PCDHGA7	P2RX1	GGT7	HLA.E	PTGER4	ANO9
SLC6A6	BTF3	PTPRG	EVC2	SERINC2	SPN
GPR15	IGF2R	IGSF3	IL2RA	DLL1	APCDD1
PRRT3	TCTN3	SLC38A1	FURIN	CNNM3	PCDHGA12
SLC22A7	SLC17A5	FGFRL1	PCDHGB6	GPR82	LTB4R
ADAM19	LRRC32	HAVCR2	MST1R	SLC46A1	GPR150

NUP210	NETO2	PDGFRB	SCARB1	CYBB	LRRC25
MFSD11	ABCC1	SLC3A2	PLXNA3	CD22	S1PR2
ADAM8	CHRNA5	C14orf132	ITGB4	RNF130	DCBLD2
SLC30A1	TM9SF2	M6PR	HEG1	TXNDC15	CCR9
SLC5A9	LMAN2	P2RY14	SLC2A6	TMEM62	EPOR
MFSD12	MCUB	ATP13A1	P2RY10	IL13RA1	PLXDC2
TM9SF4	CELSR3	NEO1	ITGB8	MPEG1	MYOF
SUSD3	LTK	PCDHGC3	CDH24	AREG	CD9
ADCY9	GPR174	FLT3	GPA33	TMEM104	TREM1
SEMA4D	SLC22A15	SLC22A4	SLC7A5	CHRNA10	SLC16A7
PLXNA2	CD69	SLAMF7	JAM3	PKHD1L1	ABCA3
SLC41A2	BSG	FCRL3	QSOX1	CDHR3	FAM171A2
SLC12A3	ITGA5	SEMA4C	C3orf35	ENPP4	PLXNC1
C19orf38	GPR132	ST7	TNFRSF10D	PIEZO1	VSIG10
ITGB7	SLC51A	LRRN1	TNFSF4	SDK2	FAM174A
SEMA4F	DCHS2	TMEM9B	FZD7	BTN3A2	SLC38A9
CD70	CRIM1	SLC1A5	ACVR2A	SLC23A1	MFSD8
NETO1	ACVR2B	SLC39A14	TSPAN17	CDHR1	PRLR
LYVE1	CSF1	ADORA2A	TNFSF8	LPAR5	CD200R1
IFNGR1	TNFRSF8	SLC16A5	CD164	ITGA7	PLAUR
ATP1B3	TMEM132A	TNFRSF1A	TTYH2	DDR2	FRRS1
HLA.C	TMEM182	ATP1A1	ABCA6	FCRL4	BACE2
ADAM23	HLA.F	SLC29A3	ADAM17	LRRC37A	SLC2A11
LMAN2L	SDK1	MCOLN1	CACHD1	SLC19A2	SLC43A2
TMPRSS15	LBH	S1PR5	NIPAL3	CLEC4G	LPAR6
SELP	ATP13A2	TMEM231	CD24	KCNMB4	GRIN2C
KEL	TSPAN13	S1PR4	SLC29A1	ITGAX	CHRNB1
LMBRD1	SLC8B1	PI16	C19orf18	SLC2A5	SLC22A5
SSR1	GPR171	CADM1	STIM1	BTN2A1	CD63
PODXL	CD52	CD151	TMEM131L	GPAT4	LAMP3
MICB	TMEM63A	SLC5A3	CD79B	JAML	GPR152
CALHM2	SNRPD1	EIF2A	CEACAM1	MS4A6A	HLA.DPB1
CD320	TCIRG1	HTR3A	SLC9A1	CELSR1	PTH2R
CPM	SUSD1	ACVR1B	SIT1	ADCY7	GPR160
DLK2	CNR1	TCTN2	LMBR1	SLC37A2	ATP6V0A2
L1CAM	FFAR1	SPPL2A	CCR5	CIP2A	SLCO4C1
ACHE	NT5E	TSPAN18	SLC38A11	KCNA3	MC1R
LRIG2	ICAM1	SLC16A6	SCARA5	CD274	UPK3B
OSTM1	TM9SF3	CALCRL	DLL4	OR52N4	SLC6A9
AMIGO2	CDHR2	ITGA4	CDH1	ATP13A3	SLC12A2
FCRL1	PTCH1	SGCA	IL2RG	TRABD2A	TLR2
ICOSLG	EDA2R	SIGLEC6	SLC2A8	ANKH	LAMP5
CD84	F2R	PRSS21	DPEP2	MFAP3	HLA.DRA
ICAM3	SMAP2	ADAM15	GRIN2D	SLC22A1	ADRA1B
EFNA3	RNF150	IL2RB	SLC29A4	PVR	TSPAN33
CCR2	SERINC1	GRIK3	ADIPOR2	ITGAE	IL7R
P2RY12	SLC12A4	TNFSF11	EFNA1	LDLR	SLC26A1
DLL3	SLC6A16	BTLA	ABCB1	UBAC2	BOC
KLRK1	GPR183	LRP6	PQLC2	FGFR1	ROR2
GPC5	PTK7	BEST4	SLC26A2	TMEM179B	TGFBR2

BRI3	IGSF9B	FNDC9	ITM2B	SCARF2	RELL1
HLA.B	BST2	PTPA	TENM4	PTTG1IP	C3orf52
PSEN1	MRC2	SUCO	IFNAR2	LRP1	GPR180
FAM174B	PTPRN2	SLC39A9	CD37	DAG1	TNFRSF18
MRVI1	CRB2	SELL	ITGA10	MAMDC4	SLC45A4
IL10RA	TNFRSF11A	INSR	C18orf54	VN1R1	FCRL2
UGT8	CD4	SLC13A4	ICAM5	GABBR1	SPNS2
LYPD6B	IL17RB	LNPEP	PDCD1	CSF1R	GPR153
CX3CL1	SORL1	EPCAM	SIGLEC14	CD83	ICAM2
CD40	SLC37A1	NAALADL1	EMP3	IL6ST	ACP2
ACE	ABCC10	SLC46A3	CD3D	LHFPL4	IGSF8
DSCAML1	PNP	GPR107	TMEM106B	CTNS	NLGN3
ADAM12	LINGO3	LRP1B	TMEM140	FCGR3A	TM9SF1
SLC9A6	LILRB2	ITGB3	SLC36A1	MICA	P2RY1
GALR2	DIRC2	LILRA5	TMCO3	PILRB	TMEM116
LILRA2	SLC35A5	SLC15A3	GPR157	AOC3	ADRB2
SUSD5	LIG1	NIPAL2	PHEX	DPP4	CD79A
TNFRSF21	TMPRSS13	IFNAR1	P2RY8	LRFN3	GABRA4
MEGF11	IL12RB2	IL11RA	VNN2	SECTM1	CD55
CLSTN3	ABCA5	CDH13	MYADM	GPR155	ERMAP
DDR1	PTCHD1	TMEM63C	FZD1	ANPEP	MR1
TGFBR1	OR2W3	SLC31A1	SLC1A3	TSPAN3	CXCR6
LRP5	CD96	ANO6	GDA	PTPRC	ROBO3
ALPL	PTPRS	NPC1	TMEM9	RNF13	IGSF6
GLIPR1	SEMA4B	SEZ6	GJB6	SCAP	MDGA1
GPR35	ABCA7	SCARB2	SLC38A2	PKD1L1	ATG9A
NOTCH1	LMBR1L	TMPRSS6	KIAA1324L	SLAMF1	GPRC5C
LRRTM2	TMEM245	HLA.G	ITFG1	SIDT2	SLC47A1
AMIGO1	TSPAN31	GPR137C	NFAM1	IL17RC	GIPR
CHL1	SLC2A13	SCN3A	KIT	MFAP3L	PTPRJ
SLCO5A1	GPR137B	P2RY11	CA14	SLC1A4	S1PR1
LGR4	RGMB	SEMA7A	TMX3	P2RX7	RNFT1
PTPRM	ADCY3	ULBP2	LRP10	BMPR2	CNTNAP2
EPHB3	AXL	PROCR	HACD4	NRCAM	FZD3
C5AR1	PTPRT	ADAM28	CLMP	IL3RA	OR2L13
UMODL1	CD1D	LRRN2	HBP1	RTN4R	SSTR3
CNR2	CCR6	TSPAN14	SLC36A4	SLC15A2	SLC20A2
SLC5A5	ZFYVE27	PANX1	KREMEN2	LRP12	SLC39A10
STS	TAS2R14	CASD1	IL1RAP	DGCR2	NUP210L
ITGB2	TMEM161A	FCGR2C	SLC11A1	LYSMD3	LAMP1
FGFR4	CACNA1C	SIRPA	OPRL1	HEPHL1	CD300C
TMEM30A	TNFRSF1B	TSPAN4	PLXNB2	AGER	LILRB3
SPPL2B	C3AR1	PODXL2	ROR1	SIGLEC10	RYK
PGAP1	PRNP	BTN2A2	CHRNE	SIRPB1	TACR1
THBD	SMO	IGHEP2	C1orf159	AMN	PKD2
CD101	FCGRT	SSTR2	RXFP4	CNNM2	P2RX6
SLAMF6	GPR37L1	SLC4A5	CD302	MEGF8	F11R
CLEC7A	STT3B	HLA.DOB	LARGE2	CD74	MPZL1
MMP25	SLC11A2	SIGLEC5	RNF43	TTYH1	NLGN4Y
APPL2	CELSR2	ABCC2	LY6E	SLC12A8	SAT1

TLR1	PSAP	ANO7	MPZL2	PLXNA1
GP6	MS4A1	GPR143	CD300LF	CDH23
TPBG	HLA.DPA1	HHLA2	CNTNAP3	F2RL2
NIPAL4	NPR2	SLC12A9	EFNB1	CLSTN1
CXCL16	DAGLB	MAG	SCN8A	CATSPERG
TMEM106A	PILRA	ALG2	SEMA6A	ABCC5
CLN3	FLVCR2	ULBP1	SLC6A12	ITGAD
CD180	CCRL2	IGSF9	TMEM204	LILRB5
HLA.H	ADAM10	HLA.DMA	NRROS	VIPR1
SLC37A3	EFNA4	TLR8	TAP2	HLA.DRB5
JAG2	SEMA4A	OXER1	FZD5	RECK
C1orf56	LEPR	ERBB2	NLGN4X	GPR22
SLC43A3	CR2	NRP2	CD6	GPR137
CLDN7	GABRR2	TLR4	FAM171A1	APC2
TMEM8B	DISP2	LY9	TNFRSF4	CD1C
SLC5A11	ITGA1	TMEM255A	TNFRSF9	LY75
ABCA2	SLC41A1	CD47	NOTCH2	LILRA4
FCGR2B	SLC29A2	PIK3IP1	SLC39A4	SLC16A4
RELT	FAM189B	PKD1	TMEM8A	TM7SF3
MILR1	CDH26	SLC3A1	TLR7	LDLRAD4
OR13A1	NPR1	SLCO4A1	MEGF9	GPR65
ACKR3	TNFRSF14	ADRB1	EPHB6	IFNGR2
CHRM4	ATRN	CD48	FNDC5	CNTNAP1
GPR161	ERVK13.1	SCARF1	SLC2A3	HTR2B
TMEM63B	SEMA5B	KCNJ3	HLA.DMB	HLA.DRB1
FZD6	ZACN	EPHB4	SCNN1D	UPK3A
VLDLR	TMEM94	MYO1C	FKRP	PIGR
NPTN	SLC44A2	CLDN12	ITM2C	HLA.DQB2
LRRC19	SLC5A6	TMEM255B	HLA.DQA1	KCNMB3
CCR7	GRIK1	IGFLR1	LRFN1	GPM6B
SORT1	NCSTN	BAMBI	OXTR	CXCR5
MFSD2B	SLC22A17	ITGA6	SPINT2	CHPT1
GYPC	LILRB1	P2RY6	ATP1A3	CLDN15
CSMD1	VASN	TMEM178B	ADCY6	HLA.DQB1
C5orf15	PTPRA	SCN2A	PIGO	DISP1
SLC12A6	NIPAL1	CD38	LRRC37B	LMBRD2
SEMA4G	KLRF1	LRRC37A3	MME	CD244
PTPRO	SLC41A3	IL10RB	IL18RAP	HLA.DQA2
CXCR4	FCGR2A	SLFN5	PDCD1LG2	GPM6A
SERINC5	PRND	TSPAN9	GRAMD1B	OMG
IGF1R	BTN2A3P	KCNJ14	GPBAR1	SLC2A14
HBEGF	SYP	TSPAN15	SLC12A7	
CLEC4M	RNF167	SPIN1	DUOX1	
LRRC37A2	SLC14A1	SPNS3	GRIN2A	
LAT2	ADORA2B	GPC1	UNC93B1	
ADAM9	PMEL	IL17RA	TMEM123	
TP53I13	CD109	LRFN4	LAMP2	
SIDT1	PCDH12	SLC10A1	P2RY13	
FLRT1	HLA.DOA	SLC22A23	SELPLG	
SLC44A5	NOTCH4	GPR3	EFNB2	

Supplementary Table 2. Information of liver allograft recipients (N=22)

Patient ID	Sex	Age	Race	Diagnosis	Immunosuppression	Sample collection year after post-transplant	DSA Status	Rejection Status
1*	M	48	White: NS/U	CCC	cyclo and mmf	2,5	positive	No
2	F	55	White: NS/U	PBC	tac, mmf, pred	5	negative	No
3*	M	58	White: NS/U	LC	rapa monotherapy	2,5	positive	No
4*	M	45	White: NS/U	LCPNCTC	tac and prednisone	2,5	positive	No
5	M	49	White: NS/U	CCB	tac monotherapy	2	positive	No
6	M	19	African American	BAE	tac, mmf, steroids	2	negative	No
7	M	45	Mexican	CC	tac monotherapy	5	negative	No
8	F	30	African American	MD	tac, mmf and pred	2	positive	Yes
9	M	55	White: NS/U	CC	tac and pred	5	positive	No
10	M	45	White: NS/U	LCPNCTC	tac monotherapy	2	positive	No
11*	M	50	Mexican	CCC	tac and MMF	2,5	positive	No
12	F	57	African American	PSC	tac, mmf, steroids	2,5	negative	No
13*	M	42	White: NS/U	Sfhf-Unsp	tac and pred	2,5	negative	No
14	M	48	White: NS/U	CCC	cyclo and MMF	5	positive	No
15*	M	52	Mexican	LC	tac monotherapy	2,5	negative	No
16*	M	50	Mexican	LCPNCTC	cyclo and rapa	2,5	negative	No
17*	M	48	White: NS/U	CCB	cyclo and mmf	2,5	negative	No
18*	F	40	White: NS/U	CALB	tac, rapa, pred	2,5	negative	No
19	M	50	White: NS/U	LCPNCTC	cyclo monotherapy	5	negative	No
20	M	61	White: NS/U	MD	rapa and mmf	5	positive	No
21	F	28	White: NS/U	Fhf-Tylenol	tac, rapa, pred	2	negative	Yes
22	F	42	White: NS/U	PBC	tac, mmf, and pred	2	negative	No

* Patient has two time point samples.

Cyclo, Cyclosporin; MMF, Mycophenolate mofetil; TAC, tacrolimus; CAM, Campath; ZEN, Zenapax; CNI, Calcineurin inhibitors (Cyclosporin/Neoral/Tac/Generic Tac/TACX); mTOR, Mechanistic target of rapamycin (Sirolimus/Everolimus); RAPA, Rapamycin; PRED, Prednisone; MPR, Methylprednisolone, HC, Hydrocortisone. CCC, Cirrhosis Chronic C; PBC, Primary Biliary Cirrhosis; LC, Laennec's Cirrhosis; LCPNCTC, Laennec's Cirrhosis & Post Necrotic Cirrhosis Type C; CCB, Cirrhosis Chronic B; BAE, Biliary Atresia: Extrahepatic; CC, Cirrhosis: Cryptogenic; MD, Metabolic Disease: Wilson'S Disease, Other Copper Disorders, PSC, Primary Sclerosing Cholangitis: No Bowel Disease; CALB, Cirrhosis: Autoimmune, Lupoid, Banti S; Fhf, fulminant hepatic failure.

Supplementary Table 3. Information of renal allograft recipients (N=20)

Patient ID	Sex	Age	Race	Diagnosis (Cause of ESRD)	Steroids/ Immunosuppression	Sample collection year after post-transplant	DSA Status	Rejection
1	M	52	European Descent	Membranous	Prograf, MMF, Pred	1	Positive	0
2	M	44	European Descent	DM-2	Prograf, MMF	1	Negative	0
3	M	43	European Descent	ADPKD	Prograf, MMF	1	Negative	0
4	F	47	European Descent	MPGN	Prograf, MMF, Pred	1	Positive	0
5	M	60	European Descent	membranous	Prograf, MMF, Pred	1	Negative	0
6	F	25	European Descent	immune complex proliferatitive GN	Prograf, MMF, Pred	1	Negative	0
7	F	42	European Descent	unknown	Prograf, MMF, Pred	1	Negative	0
8	F	57	European Descent	MPGN type 1 and DM-2	Prograf, MMF	1	Negative	0
9	M	27	European Descent	IgA	Prograf, MMF	1	Negative	0
10	M	43	European Descent	ADPKD	Prograf, MMF	1	Negative	0
11	F	27	European Descent	DM-1	Prograf, MMF, Pred	1	Positive	0
12	M	44	European Descent	ADPKD	Prograf, MMF, Pred	1	Negative	0
13	M	68	African American	MPGN	Prograf, MMF, Pred	1	Negative	0
14	M	67	European Descent	MPGN type 1 and DM-2	Prograf, MMF, Pred	1	Negative	0
15	M	44	European Descent	DM-1	Prograf, MMF, Pred	1	Negative	0
16	M	56	White: NS/U	DM-2	Prograf, MMF	1	Positive	0
17	M	62	European Descent	NSAID, AKI in setting of prostatectomy	Prograf, MMF	1	Positive	0
18	M	40	European Descent	FSGS	Prograf, MMF	1	Negative	0
19	M	63	European Descent	DM-2	Prograf, MMF	1	Negative	0
20	F	59	European Descent	ADPKD	Prograf, MMF	1	Negative	0

Following abbreviations are used: ESRD, end stage of renal diseases; DM-2, ADPKD, autosomal dominant polycystic kidney disease, MPGN, membranoproliferative glomerulonephritis; DM-1, diabetes mellitus type-1; DM-2, IgA, immunoglobulin A; AKI, acute kidney injury; diabetes mellitus type-2; NSAID, nonsteroidal anti-inflammatory drug; GN, glomerulonephritis; FSGS, focal segmental glomerulosclerosis; Prograf, tacrolimus; prednisone; MMF, mycophenolate mofeti; NS/U, not specified/unknown; Campath, Alemtuzumab.
