# Supplementary material for: Implication of TIGIT+ human memory B cells in immune regulation
Source: Nat Commun. 2021 Mar 9;12:1534. doi: 10.1038/s41467-021-21413-y (PMC7943800; doi:10.1038/s41467-021-21413-y)
Supplement: Supplementary file 3 — Reporting summary [file 41467_2021_21413_MOESM3_ESM.pdf]

nature research

Corresponding author(s): HyeMee Joo and SangKon Oh
Last updated by author(s): 12/30/2020

Reporting Summary

Nature Research wishes to improve the reproducibility of the work that we publish. This form provides structure for consistency and transparency in reporting. For further information on Nature Research policies, see our Editorial Policies and the Editorial Policy Checklist.

Statistics

For all statistical analyses, confirm that the following items are present in the figure legend, table legend, main text, or Methods section.

n/a	Confirmed	
☐	☒	The exact sample size (*n*) for each experimental group/condition, given as a discrete number and unit of measurement
☐	☒	A statement on whether measurements were taken from distinct samples or whether the same sample was measured repeatedly
☐	☒	The statistical test(s) used AND whether they are one- or two-sided<br>*Only common tests should be described solely by name; describe more complex techniques in the Methods section.*
☒	☐	A description of all covariates tested
☒	☐	A description of any assumptions or corrections, such as tests of normality and adjustment for multiple comparisons
☐	☒	A full description of the statistical parameters including central tendency (e.g. means) or other basic estimates (e.g. regression coefficient) AND variation (e.g. standard deviation) or associated estimates of uncertainty (e.g. confidence intervals)
☐	☒	For null hypothesis testing, the test statistic (e.g. *F*, *t*, *r*) with confidence intervals, effect sizes, degrees of freedom and *P* value noted<br>*Give P values as exact values whenever suitable.*
☒	☐	For Bayesian analysis, information on the choice of priors and Markov chain Monte Carlo settings
☒	☐	For hierarchical and complex designs, identification of the appropriate level for tests and full reporting of outcomes
☐	☒	Estimates of effect sizes (e.g. Cohen's *d*, Pearson's *r*), indicating how they were calculated

Our web collection on statistics for biologists contains articles on many of the points above.

Software and code

Policy information about availability of computer code

Data collection	Flow Cytometry : BD LSRFortessa, FacsDiva software (v8.0.1)
Data analysis	Flow Cytometry: FlowJo software version 10.6.2 and FacsDiva software v8.0.1, Statistical Analysis : GraphPad Prism 8.3.1 , RNA-Seq : DESec2 package ((v1.16.1), , FASTQC (v2.2.3) , hisat2 (v2.1.0), cutadapt (v1.14), samtools (v1.5), HTSeq-count (v 0.9.1).

For manuscripts utilizing custom algorithms or software that are central to the research but not yet described in published literature, software must be made available to editors and reviewers. We strongly encourage code deposition in a community repository (e.g. GitHub). See the Nature Research guidelines for submitting code & software for further information.

Data

Policy information about availability of data

All manuscripts must include a data availability statement. This statement should provide the following information, where applicable:
- Accession codes, unique identifiers, or web links for publicly available datasets
- A list of figures that have associated raw data
- A description of any restrictions on data availability

All sequencing data are available in public now.RNA-Seq data have been deposited to the Gene Expression Omnibus (GEO) GSE151415 (https://www.ncbi.nlm.nih.gov/geo/query/acc.cgi?&acc=GSE151415)

Field-specific reporting

Please select the one below that is the best fit for your research. If you are not sure, read the appropriate sections before making your selection.

[x] Life sciences [ ] Behavioural & social sciences [ ] Ecological, evolutionary & environmental sciences

For a reference copy of the document with all sections, see nature.com/documents/nr-reporting-summary-flat.pdf

Life sciences study design

All studies must disclose on these points even when the disclosure is negative.

Sample size	Samples were chosen based on availability of liver/ kidney transplant patient material. We did enroll 22 liver transplant (some of them had samples at 2 and 5 years after post-transplant) and 20 kidney transplant patients (5 DSA+ and 15 DSA-) in our study. This number of patients enabled us to find statistically significant correlations with the cell populations of interest and the parameters that we analyzed.
Data exclusions	Data were not excluded
Replication	All in vitro assays were performed at least three times with different donors. The data presented is representative of one assay. All attempts at replication were successful.
Randomization	Kidney or liver transplant patients were allocated into different experimental groups according to the presence or absence of DSA in different time points.
Blinding	All Kidney and liver transplant patient samples acquisition and analyses were performed blinded in terms of DSA status.

Reporting for specific materials, systems and methods

We require information from authors about some types of materials, experimental systems and methods used in many studies. Here, indicate whether each material, system or method listed is relevant to your study. If you are not sure if a list item applies to your research, read the appropriate section before selecting a response.

Materials & experimental systems

n/a	Involved in the study	
[ ]	[x]	Antibodies
[x]	[ ]	Eukaryotic cell lines
[x]	[ ]	Palaeontology and archaeology
[x]	[ ]	Animals and other organisms
[ ]	[x]	Human research participants
[x]	[ ]	Clinical data
[x]	[ ]	Dual use research of concern

Methods

n/a	Involved in the study	
[x]	[ ]	ChIP-seq
[ ]	[x]	Flow cytometry
[x]	[ ]	MRI-based neuroimaging

Antibodies

Antibodies used

Anti-IgD FITC (clone: IA6-2) (BD Biosciences Cat# 555778, RRID:AB_396113)
Anti-IgD Alexa Fluor 700 (clone: IA6-2)(BioLegend Cat# 348230, RRID:AB_2563335)
Anti-IgM Alexa Fluor 700 (clone: MHM-88) (BioLegend Cat# 314538, RRID:AB_2566615)
Anti-IgM APC-Cy-7 (clone: MHM-88) (BioLegend Cat# 314520, RRID:AB_10900422)
Anti-CD1c BB 700 (clone: F10/21A3) (BD Biosciences Cat# 746095, RRID:AB_2743468)
Anti-CD1c APC-Cy-7 (clone:L161)(BioLegend Cat# 331520, RRID:AB_10644008)
Anti-CD1d APC (clone: 51.1)(BioLegend Cat# 350308, RRID:AB_10642829)
Anti-CD5- APC-Cy-7 (clone:UCHT2) (BD Biosciences Cat# 563516, RRID:AB_2738249)
Anti-CD9 V450 (clone:M-L13)(BD Biosciences Cat# 561326, RRID:AB_10896331)
Anti-CD10 BV 786 (clone:HI10a) (BD Biosciences Cat# 564960, RRID:AB_2739025)
Anti-CD19 BUV 395 (clone:SJ25C1) (BD Biosciences Cat# 563549, RRID:AB_2738272)
Anti-CD19 BUV 737 (clone:SJ25C1) (BD Biosciences Cat# 612757, RRID:AB_2870088)
Anti-CD21 BV 786 (clone:1048) (BD Biosciences Cat# 742764, RRID:AB_2741029)
Anti-CD23 FITC (clone:EBVCS-5)(BioLegend Cat# 982902, RRID:AB_2650646)
Anti-CD24 PE-CF-594 (clone:ML5)(BD Biosciences Cat# 562405, RRID:AB_11153321)
Anti-CD25 BV 786 (clone:M-A251) (BD Biosciences Cat# 563700, RRID:AB_2744338)
Anti-CD27 PE (clone:M-T271)(BD Biosciences, Cat # 555441)
Anti-CD27 BV421 (clone: M-T-271)(BD Biosciences Cat# 555441, RRID:AB_395834)
Anti-CD38 PE-Cy-7 (clone:HB7)(BD Biosciences Cat# 335790, RRID:AB_399969)

Anti-CD38 Alexa Fluor 700 (clone:HIT2)(BD Biosciences Cat# 560676, RRID:AB_1727472)
Anti-CD39 BV711 (clone:TU66)(BD Biosciences Cat# 563680, RRID:AB_2738369)
Anti-CD43 BB700 (clone:L60)(BD Biosciences Cat# 746014, RRID:AB_2743409)
Anti-CD71 PE-Cy-7 (clone:CY1G4)(BioLegend Cat# 334112, RRID:AB_2563119)
Anti-CD73 BV785 (clone:AD2)(BioLegend Cat# 344028, RRID:AB_2687234)
Anti-CD73 BV605 (clone:AD2) (BioLegend Cat# 344024, RRID:AB_2650974)
Anti-CD147- PE (clone:HIM6)(BioLegend Cat# 306212, RRID:AB_2750168)
Anti-CXCR3 FITC (clone:G025H7) (BioLegend Cat# 353704, RRID:AB_10983066)
Anti-TIM-1 APC (clone:1D12) (BioLegend Cat# 353906, RRID:AB_2564325)
Anti-TIM-1BV 786 (clone:1D12) (BD Biosciences Cat# 742544, RRID:AB_2740855)
Anti-CD11a BV650 (clone:HI111) (BD Biosciences Cat# 563934, RRID:AB_2738493)
Anti-CD11b APC (clone:CBRM1/5) (BioLegend Cat# 301410, RRID:AB_2280647)
Anti-CD11c V450 (clone:B-ly6)((BD Biosciences Cat# 560369, RRID:AB_1645557)
Anti-CD45RA (clone:HI100)((BD Biosciences Cat# 555488, RRID:AB_395879)
Anti-CD49a/α1 FITC (TS2/7)(BioLegend Cat# 328308, RRID:AB_2129084)
Anti-CD49d/ α 4 BUV 737 (clone:9F10)(BD Biosciences Cat# 612850, RRID:AB_2870170)
Anti-CD29/ β1 PE-Cy-5 (clone:TS2/16) (BioLegend Cat# 303006, RRID:AB_314322)
Anti-Integrin β7 BUV 737 (clone:FIB504) (BD Biosciences Cat# 749664, RRID:AB_2873928)
Anti-PD-L1 Purified (clone:29E.2A3)(BioLegend Cat# 329702, RRID:AB_940372)
Anti-PD-L1 APC (clone:29E.2A3) (BioLegend Cat# 329708, RRID:AB_940360)
Anti-PD-L1 BV650 (clone:29E.2A3) (BioLegend Cat# 329740, RRID:AB_2629614)
Anti-PD-L1BV711 (clone:29E.2A3) (BioLegend Cat# 329722, RRID:AB_2565764)
Anti-TIGIT BV605 (clone:A15153G) BioLegend Cat# 372712, RRID:AB_2632927)
Anti-TIGIT Purified (clone:A15153G) (BioLegend Cat# 372702, RRID:AB_2632714)
Anti-TIGIT Purified (clone:MBSA43) (Thermo Fisher Scientific Cat# 16-9500-82, RRID:AB_10718831)
Anti-TIGIT PE (clone:A15153G) (BioLegend Cat# 372704, RRID:AB_2632730)
Anti-IL-10 Purified (clone:JES3-19F1)(BioLegend Cat# 506802, RRID:AB_315452)
Anti-IL-10 eFluor 450 (clone:JES3-9D7) (Thermo Fisher Scientific Cat# 48-7108-42, RRID:AB_10548941)
Anti-IL-10 Alexa Fluor 647 (clone:JES3-9D7) (BioLegend Cat# 501412, RRID:AB_493318)
Anti-IL-6 FITC (clone:MP5-20F3) (Thermo Fisher Scientific Cat# 11-7061-41, RRID:AB_1633408)
Anti-IL-6 PerCP-eFluor 710 (clone:MQ2-13A5) (Thermo Fisher Scientific Cat# 46-7069-42, RRID:AB_11151511)
Anti-TNFα PE-Cy-7 (clone:MAb11)(BioLegend Cat# 502930, RRID:AB_2204079)
Anti-TNFα APC (clone:MAb11)((BioLegend Cat# 502912, RRID:AB_315264)
Anti-TNFα BV421 (clone:MAb11)((BioLegend Cat# 502932, RRID:AB_10960738)
Anti-IFNγ PE-Cy-7 (clone:4S.B3)(BioLegend Cat# 502528, RRID:AB_2123323)
Anti-IFNγ Alexa Fluor 700 (clone:4S.B3) (BioLegend Cat# 502520, RRID:AB_528921)
Anti-IFNγ BV 785 (clone:4S.B3)(BioLegend Cat# 502542, RRID:AB_2563882)
Anti-IL-17 Alexa Fluor 700 (clone:BL168) (BioLegend Cat# 512318, RRID:AB_2124868)
Anti-IL-17 BV 605 (clone:BL168) (BioLegend Cat# 512326, RRID:AB_2563887)
Anti-CD4 APC-Cy-7 (clone:RPA-T4) (BioLegend Cat# 300518, RRID:AB_314086)
Anti-CD4 APC (clone:RPA-T4) (BioLegend Cat# 300514, RRID:AB_314082)
Anti-CD8 BV 711 (clone:RPA-T8)(BioLegend Cat# 301044, RRID:AB_2562906)
Anti-CD8 BV 570 (clone:RPA-T8) (BioLegend Cat# 301038, RRID:AB_2563213)
Anti-CD3 BUV 737 (clone:UCHT1)(BD Biosciences Cat# 612750, RRID:AB_2870081)
Anti-CD3 APC (clone:UCHT1) (BD Biosciences Cat# 555335, RRID:AB_398591)
Anti-Granzyme B Alexa Fluor 700 (clone:GB11) (BD Biosciences Cat# 561016, RRID:AB_2033973)
Anti-TGFβ1 Purified (clone:TW7-28G11) (BioLegend Cat# 146704, RRID:AB_2562710)
Anti-TGFβ1-PE (clone:TW4-6H10) (BioLegend Cat# 349704, RRID:AB_10639862)
Anti-IL-10R Purified (clone:3F9)(BioLegend Cat# 308802, RRID:AB_314734)
Anti-CD80 APC-H7 (clone:L307.4)(BD Biosciences Cat# 561134, RRID:AB_10565974)
Anti-CD83 PE-CF-594 (clone:HB15e) (BD Biosciences Cat# 562631, RRID:AB_2737688)
Anti-CD86 BV 650 (clone:IT2.2) (BioLegend Cat# 305428, RRID:AB_2563823)
Anti-CD40 FITC (clone:5C3) (BD Biosciences Cat# 555588, RRID:AB_395963)
Anti-CCR7 BV 711 (clone:3D12)(BD Biosciences Cat# 563712, RRID:AB_2738386)
Anti-CCR7 PE-Cy-7 (clone:3D12)(BD Biosciences Cat# 560922, RRID:AB_10561680)
Anti-ICOS-L PE-Cy-7 (clone:2D3) (BioLegend Cat# 309410, RRID:AB_2565671)
Anti-HLA DR Alexa Fluor 700 (L243)(BioLegend Cat# 307626, RRID:AB_493771)
Anti-IL-12A PE (clone: 2Y37)(Novus Biological, Cat # NBP2-22038PE, RRID: n/a)
Anti-CD155 PE-Cy-7 (clone:SKII.4) (BioLegend Cat# 337614, RRID:AB_2565747)
Anti-CD127 BV 421 (clone:A019D5)(BioLegend Cat# 351310, RRID:AB_10960140)
Anti-ICOS PE-Cy-7 (clone:C398.4A) (BioLegend Cat# 313520, RRID:AB_10643411)
Anti-CXCR5 BV 711 (clone:RF8B2)(BD Biosciences Cat# 740737, RRID:AB_2740408)
Human cell surface marker screening kit (BD Biosciences, Cat # 560747)
Anti-IL-21 PE (clone:3A3-N2.1)(BD Biosciences Cat# 560463, RRID:AB_1645516)
Anti-IL-4 FITC (clone:MP4-25D2)(BD Biosciences Cat# 554484, RRID:AB_395423)

Validation	All antibodies are established, well described, and published elsewhere. Informations are accessible on the manufacturers websites under Cat # or RRID numbers.

Human research participants

Policy information about studies involving human research participants

Population characteristics	In liver transplant patient cohort's included diverse background (Race: white, African American, and Mexican; Age ranges : 48-61; gender: 66 % male and 34% female). In kidney transplant cohort's , majority patients were from European decent , one from African American, and one White. All kidney transplant patient blood's collected one year after post transplant. More details can be found Supplemental table 2 and 3.
Recruitment	Both liver and renal allograft recipients were recruited by clinician and blood samples from transplant patients were collected with appropriate consents at prespecified time points post-transplant and/or at the time of regular biopsies. Anti-HLA IgG antibodies were used to detect donor specific allo-antibodies (DSA) of all patients and separated them DSA+ or DSA- group. Healthy subjects were selected random by picking people with matching age and gender
Ethics oversight	All experiments using human samples were performed in accordance with the protocols approved by the Institutional Review Board (IRB) in Mayo Clinic and Baylor Scott & White Research Institute. This work was carried out in accordance with the Code of Ethics of the World Medical Association (Declaration of Helsinki). Blood samples from transplant patients were collected with appropriate consents at prespecified time points post-transplant and/or at the time of regular biopsies.

Note that full information on the approval of the study protocol must also be provided in the manuscript.

Flow Cytometry

Plots

Confirm that:

- [x] The axis labels state the marker and fluorochrome used (e.g. CD4-FITC).
- [x] The axis scales are clearly visible. Include numbers along axes only for bottom left plot of group (a 'group' is an analysis of identical markers).
- [x] All plots are contour plots with outliers or pseudocolor plots.
- [x] A numerical value for number of cells or percentage (with statistics) is provided.

Methodology

Sample preparation	Peripheral blood mononuclear cells (PBMCs) and mononuclear cells (MNCs) from tonsils were isolated by density gradient centrifugation using Ficoll-Paque PLUS (GE Healthcare). B cells were purified by negative selection using a pan-B cell enrichment kit (STEMCELL Technologies). Indicated subsets of B cells were sorted with a BD FACSAria II (BD Biosciences).
Instrument	BD FascAria II, BD LSR Fortessa,
Software	BD FACSDiva Software (v8.0.1) were used for flow data collection. Flow cytometry data were analyzed with FlowJo v. 10.6.3.
Cell population abundance	Cell subsets were sorted with >98% purity as controlled by remeasurement of cell sorted population
Gating strategy	Populations as indicated in figure legends were identified by mononuclear cells gate in FSC-A/SSC-A, doublet exclusion by FSC-A/FSC-H, dead cell exclusion by Live/Dead Aq/SSC-A, lymphocyte gate in FSC-A/SSC-A on live cells. LIN (CD3, CD14, CD56) negative, CD19+ cells were selected for further dividing the B cell subsets. CD19+CD24hiCD38hiIgD+(P1), CD19+CD27+CD39hiIgD+ (P2), CD19+CD27-CD39+IgD+(P3), CD19+CD27+CD39hiIgD- (P4), CD19+CD27-CD39-IgD+ (P5), and CD19+CD27-CD39+ IgD- (P6) B cell subsets were sorted with a BD FACSAria II (BD Biosciences). TIGIT+ B cells and naive CD4+ T cells sorting were showed in Supplementary Fig.12 and Supplementary Fig.14, respectively

- [x] Tick this box to confirm that a figure exemplifying the gating strategy is provided in the Supplementary Information.
